# A novel HIV-1 restriction factor that is biologically distinct from APOBEC3 cytidine deaminases in a human T cell line CEM.NKR

**DOI:** 10.1186/1742-4690-6-31

**Published:** 2009-04-03

**Authors:** Tao Zhou, Yanxing Han, Ying Dang, Xiaojun Wang, Yong-Hui Zheng

**Affiliations:** 1 Department of Microbiology & Molecular Genetics, Michigan State University, East Lansing, MI 48824-4320, USA

## Abstract

**Background:**

Isolation of novel retroviral restriction factors will open new avenues for anti-HIV/AIDS treatment. Although HIV-1 replication is restricted by APOBEC3G/APOBEC3F, TRIM5α, and CD317, none defend HIV-1 infection under natural conditions. Previously, we demonstrated a host factor from the human T cell line CEM.NKR that potently restricted wild-type HIV-1 replication. Interestingly, this restriction resembled the APOBEC3G/APOBEC3F pattern in that viral replication was inhibited from the second round of replication cycle at a post-entry step.

**Results:**

Here, we further characterized this factor and found it distinguishable from the known anti-HIV APOBEC3 proteins. Although CEM.NKR cells expressed both APOBEC3G and APOBEC3F, their levels were at least 10 or 4-fold lower than those in H9 cells, and importantly, Vif effectively neutralized their activity. Among eight subclones isolated from CEM.NKR cells, one was relatively permissive, four were semi-permissive, and three were completely non-permissive for HIV-1 replication. When the levels of APOBEC3 expression were determined, all these clones retained similar low levels of APOBEC3DE, APOBEC3F, APOBEC3G and APOBEC3H expression, and no APOBEC3B expression was detected. Since the *vif *from SIVmac can effectively neutralize APOBEC3B and APOBEC3H, recombinant HIV-1 expressing this SIV gene were created. However, these viruses still failed to replicate in CEM.NKR cells. We also confirmed that HIV-1 restriction in CEM.NKR was not due to a loss of calnexin expression.

**Conclusion:**

Taken together, these results not only demonstrate that all these aforementioned anti-HIV APOBEC3 proteins do not contribute to this HIV-1 restriction, but also shed light on a novel and potent HIV-1 inhibitor in CEM.NKR cells.

## Background

CEM is a human T lymphosarcoma cell line isolated from an infant female patient with acute leukemia [1]. This human T cell line has been useful in HIV research because of its infectability and has significantly contributed to our understanding of innate intracellular immunity to retroviruses. Human T cell lines have been classified as either permissive or non-permissive cells based on their ability to support *vif*-deficient HIV-1 replication. CEM and H9 are non-permissive cell lines, whereas Sup-T1 and Jurkat are permissive lines [2,3]. Derivative cell lines have been isolated from CEM by various methods, including CEM-SS [4], CEM-T4, A3.01 [5], and CEM.NKR [6]. Interestingly, both CEM-SS and CEM-T4 are permissive for *vif*-deficient HIV-1 replication whereas A3.01 is semi-permissive [7], suggesting that the original CEM cells are quite heterogeneous. Importantly, genetic analysis of the difference between CEM and CEM-SS has led to the discovery of APOBEC3G (A3G) as one of the cellular targets of Vif [8].

A3G belongs to a small group of proteins in the cytidine deaminase family known as the APOBEC3 (A3) subfamily [9]. This group of proteins includes A3A, A3B, A3C, A3DE, A3F, A3G, and A3H. All have antiretroviral activities against different targets including exogenous retroviruses and endogenous retroelements [10]. A3B, A3DE, A3F, and A3G contain two Zinc-binding motifs, while A3A, A3C, and A3H contain only one. A3B, A3DE, A3F, and A3G inhibit HIV-1 replication to different degrees, whereas A3A and A3C do not [8,11-16]. Recently, it was shown that A3H also inhibits HIV-1 replication if its expression is optimized in cell culture [17-20]. Among these proteins, the anti-HIV activity of A3G and A3F is the most prominent. Nevertheless, HIV-1 is able to elude this defense mechanism and cause human disease for two reasons. First, A3B and A3H are poorly expressed in vivo [13,17,21,22]. Second, HIV-1 produces Vif, which binds to and mediates the destruction of A3DE, A3F, and A3G in 26S proteasomes via recruitment of the Cullin5 ubiquitin E3 ligase [23,24]. Vif may also inhibit A3 activity independent of proteasomal degradation [25-27]. In addition to Vif activity, HIV-1 replication can also be inhibited by two other types of restriction factors: TRIM5α which blocks viral uncoating [28] and cell surface protein CD317 which blocks viral release [29]. However, human TRIM5a does not inhibit HIV-1, and the antiviral activity of CD317 is neutralized by another viral protein Vpu.

CEM.NKR is a naturally isolated cell clone from CEM that is resistant to natural killer (NK) cell-mediated lysis [6]. Previously, we tried to infect CEM.NKR cells and found that they were highly resistant to productive infection by wild-type HIV-1 [30]. Further analyses indicated that CEM.NKR expressed a viral inhibitor, which did not target incoming viruses but blocked HIV-1 at the second round of replication at a post-entry step. Since this inhibitor showed a similar inhibition profile as A3G/A3F except that it inhibited the wild-type virus, we wondered whether this resistance was simply due to an over-expression or expression of a genetic variant of known A3 cytidine deaminases which could not be inhibited by Vif. Here, we present several different lines of evidence to demonstrate that this inhibitor activity is indeed novel and distinguishable from any of A3 proteins.

## Results and discussion

### Over-expression of A3G/A3F is not responsible for wild-type HIV-1 restriction in human T cells

**Figure 1:**
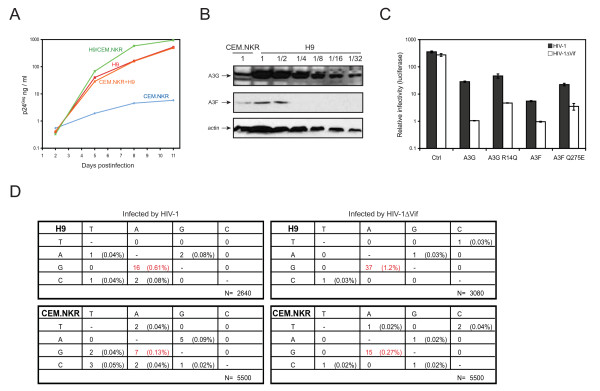
**Characterization of inhibitory activity of CEM.NKR**. A) CEM.NKR cells do not secrete a soluble HIV inhibitory factor. Either 2 × 10^5 ^H9 or CEM.NKR cells were infected with 150 ng p24^Gag ^of HIV-1 (NL4-3) for 3 hours at 37°C individually. After extensive washing, they were either cultured in separated wells, co-cultured by mixing them in the same well (CEM.NKR+H9) at 1:1 ratio, or co-cultured in the same well at 1:1 ratio separated by a insert with a 1.0 μm transparent PET membrane (H9/CEM.NKR) in 24-well plates (BD Biosciences). Viral production was determined by p24^Gag ^ELISA over an 11-day period. B) CEM.NKR cells express lower levels of A3G and A3F than H9. Cytosolic fractions were prepared from equal numbers of CEM.NKR and H9 cells and the cytosol from H9 was subjected to a two-fold serial dilution. The levels of A3G and A3F expression were analyzed by Western blotting. The levels of actin contained lysate from 5 × 10^6 ^cells (undiluted lanes) served as an internal control. C) A3G R14Q and A3F Q275E mutants retain anti-HIV-1 activity. The cDNAs of A3G R14Q and A3F Q275E were amplified from CEM.NKR by RT-PCR and cloned into pcDNA3.1/V5-His-TOPO vector (Invitrogen). Wild-type or *vif*-defective HIV-1 (NL4-3) were produced from 293T cells in the presence of these wild-type or mutant A3G or A3F proteins and viral infectivity was determined in GHOST cells. *Ctrl*, control vector (pcDNA3.1). D) Vif effectively inactivates A3G/A3F in CEM.NKR cells. H9 and CEM.NKR cells were infected with HIV-1 or *vif*-deficient HIV-1 (NL4-3) expressing a neomycin-resistant gene in the *nef *gene locus and infected cells were selected by G418 treatment. HIV genes from nucleotides 5693 to 5912 corresponding to pNL4-3 sequence were amplified from these infected cells by PCR. After cloning into TA-cloning vector, multiple clones were collected for nucleotide sequencing. The type of mutation is summarized in tabular form, where the original HIV-1 sequence is given at left, and the new sequence is given across the top. N at the lower right of each box indicates the total numbers of bases sequenced.

To further understand the mechanism of HIV-1 restriction in CEM.NKR, we first determined whether CEM.NKR cells secreted a soluble factor that inhibited HIV-1 replication. Since H9 cells can be productively infected by wild-type HIV-1, we set up a co-culture system between H9 and CEM.NKR to address this issue. After a brief incubation with wild-type virus, infected H9 or CEM.NKR cells were co-cultured by either mixing together (CEM.NKR+H9) or separated by a permeable membrane (H9/CEM.NKR) in a 24-well plate and viral production assayed as p24^Gag ^synthesized was monitored for 11 days. H9 cells alone were productively infected with peak viral production at ~500 ng/ml p24^Gag^, whereas CEM.NKR cells alone were poorly infected with maximal viral production at ~5 ng/ml p24^Gag ^(Fig. 1A). These results were consistent with our previous observations [30]. In addition, H9/CEM.NKR and CEM.NKR+H9 co-cultures were both productively infected, and viral production from the H9/CEM.NKR co-culture was slightly higher than that of H9 alone or the CEM.NKR+H9 co-culture (Fig. 1A). This result indicated that CEM.NKR cells did not secrete a soluble HIV-1 inhibitor.

Second, we compared A3G/A3F protein levels in H9 and CEM.NKR cells to determine whether A3G/A3F were overly expressed in CEM.NKR cells. Cell lysates were prepared from a total of 1 × 10^7 ^H9 or CEM.NKR cells. Two-fold serial dilutions were analyzed by Western blotting using A3G or A3F-specific antibody. Actin served as a loading control. Surprisingly, the levels of both A3G and A3F in CEM.NKR cells were around 10 or 4-fold lower than those in H9 cells, respectively (Fig. 1B). This result indicated that restriction of HIV-1 replication in CEM.NKR was not due to over-expression of A3G and A3F. We next determined whether A3G or A3F genes contained mutations that would reduce their sensitivity to Vif and contribute to the HIV-1 non-permissiveness in CEM.NKR. A3G and A3F cDNAs were amplified from CEM.NKR by RT-PCR and cloned for sequencing. Interestingly, it was found that the A3G gene contained a R14Q and A3F contained a Q275E mutation. To test how these mutations affected A3G and A3F, these genes were cloned into pcDNA3.1 assayed for their effect on HIV-1 in a single round replication cycle. Wild-type and *vif*-deficient HIV-1 luciferase reporter viruses were produced from 293T cells in the presence of the wild-type or mutant A3G or A3F expression vectors. Equal amounts of viruses were collected to infect GHOST cells and viral infectivity was determined by measuring cellular luciferase activities. Compared to the infectivity of the control HIV-1 produced in the absence of any A3G/A3F proteins, all A3G and A3F proteins (wild-type and mutants) reduced the HIV-1ΔVif virus infectivity significantly more than HIV-1 virus infectivity (Fig. 1C). For example, A3G, A3G R14Q, A3F, or A3F Q275E reduced HIV-1 infectivity by 12, 8, 60, or 16-fold and HIV-1ΔVif infectivity by 260, 60, 270, or 80-fold respectively. Although these results might indicate that A3G R14Q and A3F Q275E had slightly lower anti-HIV-1 activity, these proteins were still sensitive to Vif.

Third, we wanted to directly demonstrate that Vif could neutralize A3G/A3F in CEM.NKR cells. Our strategy was to create HIV-1 and HIV-1ΔVif virus-infected CEM.NKR cells and compare levels of G-to-A mutations in HIV-1 genomes. If the HIV-1 contained lower levels of mutations than the HIV-1ΔVif, it should further confirm A3G/A3F sensitivity to Vif. Since CEM.NKR cells were very difficult to infect, they were infected with HIV-1 or HIV-1ΔVif viruses expressing a neomycin-resistance gene (pNL-Neo, pNL-NeoΔVif) and infected cells were amplified by G418 treatment. As controls, H9 cells were infected and treated similarly. After that, cellular DNAs were extracted and HIV genes were amplified by PCR and cloned into a T-A cloning vector for sequencing. It was found that all viral genomes contained much higher rates of G-to-A of mutations than any other mutations (Fig. 1D). Further analyses found that these excessive G-to-A mutations occurred in both GG and GA dinucleotides (data not shown), which are preferred mutation sites for A3G and A3F. In addition, the G-to-A hypermutation rate was much higher in H9 than CEM.NKR cells, which was consistent with the higher A3G/A3F expression levels in H9 cells. Moreover, when Vif was present, this G-to-A hypermutation rate was reduced to half in both H9 and CEM.NKR cells (Fig. 1D). These results clearly indicated that Vif could neutralize A3G/A3F activity in CEM.NKR cells as efficiently as in H9 cells, and they also pointed out that a complete elimination of their activities by Vif might not be possible in these human T cells. Thus, we concluded that HIV-1 resistance in CEM.NKR was not due to the inability of Vif to neutralize A3G/A3F.

### Analysis of HIV-1 resistance in CEM.NKR subpopulations

**Figure 2:**
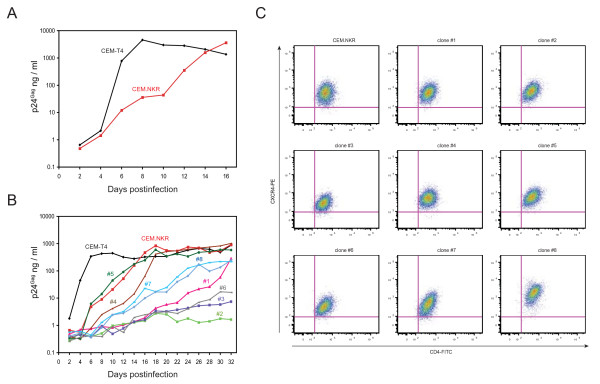
**HIV-1 resistance in CEM.NKR subpopulations**. A) HIV-1 (NL4-3) replication in CEM.NKR and CEM-T4 cells over a 16-day period. B) HIV-1 replication in cell clones from CEM.NKR. CEM.NKR cells were subjected to limiting dilution and eight cell clones (#1 to #8) were isolated. Together with CEM-T4 and the parental CEM.NKR, these clones were infected with HIV-1 (NL4-3) and viral production was measured for 32 days. C) CD4 and CXCR4 surface expression in cell clones from CEM.NKR. The parental or each individual cell clone was stained with fluorescence-conjugated antibodies (CD4-FITC, CXCR4-PE) and analyzed by flow cytometry.

Although we knew that CEM.NKR cells were resistant to productive HIV infection, we also found that they could become productively infected if they were exposed to prolonged viral infection (Fig. 2A). After 10 days, viral production jumped from lower than 100 to 1000 ng/ml p24^Gag^, which was similar to levels of viral production in CEM-T4 cells. Although CEM.NKR was reported as a cloned cell line [6], we suspected that its cell population might be heterogenous. A limiting dilution assay was used to assess CEM.NKR homogenicity. Eight clones were isolated which were subsequently inoculated with HIV-1 and cultured for 32 days. It was found that all clones as well as the parental CEM.NKR showed delayed viral replication kinetics compared to the highly permissive CEM-T4 cell lines [7]. However, using 100 ng/ml of p24^Gag ^as a criterion for productive infection, we could divide these clones into three groups based on how long it took to become productively infected (Fig. 2B). For example, clone #5 as well as the parental CEM.NKR cells were designated as permissive cells since they only took 10 days to become productively infected; clones #4, #7, #8, and #1 cells were designated as semi-permissive cells since they took 16 to 28 days to become productively infected; and clones #2, #3, and #6 cells were designated as non-permissive cells since they were not productively infected even after 32 days. It was confirmed that all these clones expressed comparable levels of CD4 and CXCR4 as the parental cell line, although clone #2 and #8 expressed higher levels of CXCR4 (Fig. 2C). Thus, CEM.NKR cells contained a subset of cells that were relatively permissive for HIV-1 replication.

### Lack of correlation of A3 expression with HIV-1 replication in CEM.NKR cell clones

**Figure 3:**
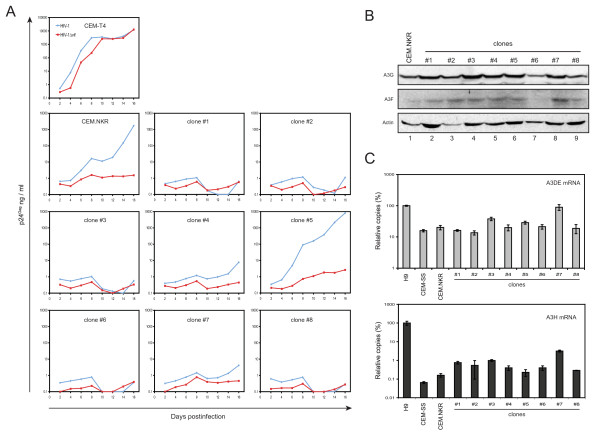
**Activity and expression of A3 cytidine deaminases in cell clones from CEM.NKR**. A) Wild-type and *vif*-defective HIV-1 replication in CEM.NKR cell subpopulations. A total of 2 × 10^5 ^of CEM-T4, CEM.NKR, and the eight clones were infected with 150 ng p24^Gag ^of wild-type or *vif*-defective HIV-1 (NL4-3) and viral production was measured for 16 days. B) A3G and A3F protein expression in CEM.NKR and its cell clones determined by Western blotting. C) A3DE and A3H mRNA expression in CEM.NKR and its cell clones as determined by real-time PCR. Results are presented as relative values, where expression levels in H9 cells are set as 100%.

Since we obtained several CEM.NKR clones that could be productively infected by HIV-1, we next compared wild-type and *vif*-deficient HIV-1 replication to understand how A3 cytidine deaminases function in these cells. As we reported previously, wild-type and *vif*-deficient HIV-1 replicated equally well in CEM-T4 cells [7] (Fig. 3A). Consistently, wild-type HIV-1 started productive replication in parental CEM.NKR and clone #5 from 8 to 10 days postinfection, and in clones #4 and #7 from 16 days postinfection. Notably, the *vif*-deficient HIV-1 failed to replicate in all these cell lines. This result further confirmed that Vif could effectively neutralize A3G/A3F proteins in CEM.NKR as well as its derived cell clone #4, #5, and #7. To further address whether the HIV-1 resistance in the other non-permissive cell clones was due to an over-expression of any A3 cytidine deaminases, we measured protein expression levels of A3G and A3F by Western blotting and mRNA expression levels of A3B, A3DE, and A3H by real-time PCR. It was found that all these cell clones except clone #6 expressed similar levels of A3G and A3F as the parental CEM.NKR (Fig. 3B). Our real-time PCR analysis failed to detect A3B in any of these cell clones as well as in H9, CEM-SS, and the parental CEM.NKR, confirming that A3B is poorly expressed in vivo. The mRNA levels of A3DE were quite similar in CEM-SS, parental CEM.NKR, and these cell clones, except that levels of A3DE mRNA in H9 and clone #7 were approximately 5-fold higher (Fig. 3C). In addition, the mRNA levels of A3H in CEM-SS, CEM.NKR, and these cell clones were 100 to 1000-fold lower than those in H9 except that clone #7 expressed slightly higher A3H mRNA (Fig. 3C). Since clones #6 and #7 exhibited a non-permissive or semi-permissive phenotype for HIV-1 infection (Fig. 2B), respectively, we therefore further confirmed that levels of A3 expression did not correlate with HIV-1 resistance in CEM.NKR and its subclones.

### Calnexin does not rescue HIV-1 replication in CEM.NKR cells

**Figure 4:**
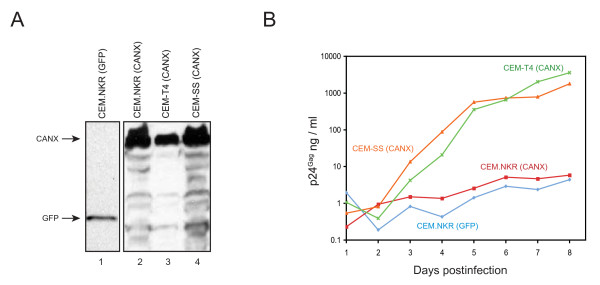
**HIV-resistance in CEM.NKR is not due to the loss of calnexin expression**. A) Transduction of calnexin gene into CEM.NKR cells. Calnexin (CANX) or a control (GFP) gene with a 3'-HA tag was cloned into the pMSCVneo retroviral vector. Recombinant retroviruses were produced by transfection of the Phoenix-AMPHO cell line and were used to infect CEM.NKR, CEM-T4, and CEM-SS cells. Four stable cell lines were created by G418 selection and the expressions of transduced genes were determined by Western blotting using an anti-HA antibody. B) HIV-1 (NL4-3) replication in these four cell lines.

It was reported that CEM.NKR cells lose a cellular protein called calnexin (CANX), a type I transmembrane protein that plays a role in the retention of misfolded glycoproteins in the endoplasmic reticulum (ER) [31]. CANX is found to interact with the uncleaved HIV-1 gp160 glycoprotein [32], and it was speculated that CANX might function as a gp160 chaperone that could play a role during viral entry [33]. To understand whether HIV restriction in CEM.NKR was due to the loss of CANX, CEM.NKR cells were transduced with an exogenous *CANX *gene containing a 3'-HA tag expressed from a retroviral vector pMSCVneo and stably transduced cells were obtained by G418 selection. Using the same procedure, CEM.NKR cells were also transduced with a green fluorescence protein (GFP) gene, and CEM-T4 and CEM-SS cells were transduced with the same *CANX *gene, were used as controls. CANX and GFP proteins were all expressed in these cells as determined by Western blotting (Fig. 4A). When these cell lines were infected with HIV-1, it was found that CEM.NKR cells expressing GFP and CANX were almost equally resistant to viral infection, whereas CEM-SS and CEM-T4 cells expressing CANX were productively infected (Fig. 4B). This result demonstrated that expression of CANX could not rescue HIV replication in CEM.NKR and excluded that the blockade of viral entry was an explanation for HIV-1 restriction in our previous observation [30].

### CEM.NKR cells express a HIV-1 inhibitor

Previously, we demonstrated that CEM.NKR cells expressed a HIV-1 inhibitor using a cell fusion assay [30]. In this assay, 293T cells expressing HIV-1 gp160 were co-cultured with CEM.NKR cells producing *env*-defective HIV-1. Only heterokaryons formed by gp160 and CD4/CXCR4 mediated cell fusion could release infectious particles, which could be detected by infection of the HIV indicator cell line TZM-BI. The levels of infectious particles from heterokaryons should positively correlate with HIV-1 resistance of the tested cell line. Notably, the levels of infectious particles from heterokaryons should also positively correlate with the fusion efficiency between 293T and the target T cells. We therefore assessed the fusion efficiency of 293T with CEM.NKR as well as its subclones.

**Figure 5:**
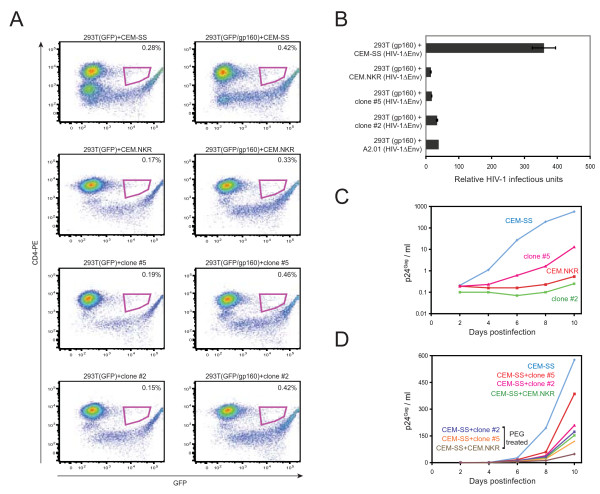
**Infectivity of HIV particles from cells fused with CEM.NKR cell clones**. A) Fusion efficiency between CEM.NKR and 293T cells. 293T cells were transfected with a GFP expression vector in the presence or absence of gp160 expression vector and then co-cultured with CEM-SS, CEM.NKR, clone #2, or clone #5, respectively. After 48 hours, cells were stained with CD4-PE and the amounts of CD4 and GFP double positive cells were measured by flow cytometry. B) Production of infectious particles from heterokaryons between 293T and CEM.NKR or its subclones. The gp160-expressing 293T cells were co-cultured with indicated T cells producing *env*-defective HIV-1. After 48 hours, production of infectious particles from these co-cultures was determined by infection of TZM-BI cells. C) HIV-1 (NL4-3) replication in CEM-SS, CEM.NKR, clone #2, and clone #5 cells. D) HIV-1 (NL4-3) replication in CEM-SS and CEM.NKR co-cultures. A total of 5 × 10^5 ^HIV-infected CEM-SS, CEM.NKR, clone #2, or clone #5 cells were co-cultured at 1:1 ratio with or without a 5 min PEG treatment. Viral production from these cultures was then determined for 10 days.

293T cells were cotransfected with GFP and gp160 expression vectors, and co-cultured with CEM-SS, CEM.NKR, clone #2, or clone #5 cells producing *env*-defective HIV-1, respectively. As controls, 293T cells were only transfected with a GFP expression vector and used in these co-cultures. The number of CD4 and GFP double positive cells were then measured by flow cytometry. The fusion efficiency was calculated by the levels of double positive cells in the co-cultures expressing gp160 after deduction from those controls. Accordingly, CEM-SS, CEM.NKR, clone #5, and clone #2 had 0.15%, 0.16%, 0.27%, and 0.27% fusion efficiency with 293T cells (Fig. 5A). Thus, CEM.NKR, clone #2, and clone #5 all had higher fusion efficiency with 293T than CEM-SS under this condition.

We next determined the levels of infectious particles produced from these co-cultures. A2.01 cells were used as a negative control because this cell line did not express CD4 and could not fuse with 293T cells expressing gp160 [7]. Indeed, co-cultures of 293T with A2.01, CEM.NKR, clone #2, and clone #5 all produced very low levels of infectious particles, whereas the co-culture of 293T with CEM-SS produced 10 to 20-fold more infectious particles (Fig. 5B). These results indicated that poor production of infectious particles from co-cultures of 293T with CEM.NKR and its subclones was not due to the poor fusion efficiency, and instead, it was due to the presence of an HIV-1 inhibitor.

To further confirm these observations, we tried to directly fuse these CEM.NKR clones with CEM-SS cells to see whether viral production could be suppressed. It is known that T-cell tropic HIV-1 can induce cell fusion and cause syncytium-formation during infection. CEM-SS cell line is not only highly permissive for HIV-1 infection, but also sensitive to HIV-1-induced syncytium-formation [4]. Thus, fusion between CEM-SS and CEM.NKR cells could occur naturally if they were co-cultured during HIV-1 infection. In addition, we tired to further increase this fusion efficiency by treating cells with polyethylene glycol (PEG).

Although clone #5 was the most permissive for HIV-1 infection among the CEM.NKR clones, we found that this cell line was still much less permissive than CEM-SS (Fig. 5C). Thus, this cell line might still express the same HIV-1 inhibitor, albeit at a decreased level. We therefore used both clone #5 and #2 for this determination. HIV-1-infected CEM-SS, CEM.NKR, clone #5, or clone #2 cells were co-cultured with or without a brief PEG treatment and viral production was determined over a 10-day period. Even without PEG treatment, levels of virus production were all reduced from CEM-SS+CEM.NKR, CEM-SS+clone #5, and CEM-SS+clone #2 co-cultures, and importantly, the reduction from CEM-SS+clone #2 was much more significant (Fig. 5D). Since we already demonstrated that CEM.NKR cells did not secrete a soluble HIV-1 inhibitor (Fig. 1A), such reduction could be explained by the appearance of cell fusion of CEM-SS with CEM.NKR, clone #5, and clone #2 cells, resulting in an inhibition of HIV-1 replication. When treated with PEG, viral production was further reduced, which could be due to an increase in cell fusion efficiency (Fig. 5D). These results not only further supported our previous conclusion that CEM.NKR cells expressed a HIV-1 inhibitor, but also indicated that clone #5 could express relatively lower levels of this inhibitor than clone #2.

### The replication of HIV-1 expressing *vif *from SIVmac is still restricted in CEM.NKR cells

It is known that *vif *from HIV-1 does not neutralize human A3B and A3H [11,17]. However, *vif *from SIVmac neutralizes not only human A3DE, A3F, and A3G, but also human A3B and A3H [11,12,17,34-36]. These observations suggest that *vif *from SIVmac is a powerful tool to knock down all these known anti-HIV human APOBEC3 proteins. Although we already knew that CEM.NKR cells had low levels of A3B and A3H expression (Fig. 3C), we wanted to further exclude that they were not responsible for HIV-1 restriction in CEM.NKR cells.

**Figure 6:**
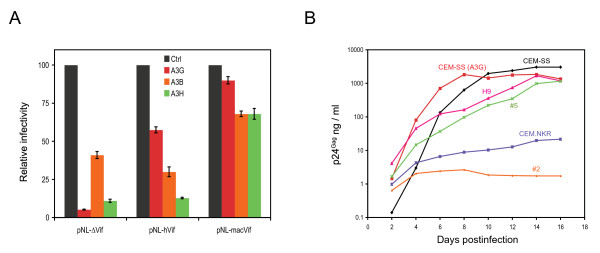
**Restriction of HIV-1 expressing *vif *fromSIVmac in CEM.NKR cells**. A) SIVmac *vif *effectively neutralized human APOBEC3 proteins in single cycle HIV-1 replication assay. Proviral constructs pNL-ΔVif, pNL-hVif, or pNL-macVif were co-transfected with pcDNA3.1 (Ctrl), pcDNA-A3G, pcDNA-A3B or VR-A3H into 293T cells at 1:1 ratio. After normalized by p24^Gag^, equal amounts of viruses were used to infect TZM-BI cells, and viral infectivity was determined by measuring cellular luciferase activity 24 hours postinfection. Results were shown as relative values, where the infectivity of viruses produced in the presence of pcDNA3.1 was set as 100. B) Replication of HIV-1 expressing *vif *from SIVmac in different human T cell lines. A total of 2 × 10^5 ^cells from indicated cell lines were infected with 150 ng p24^Gag ^of HIV-1 from pNL-macVif, and viral replications were determined for 16 days.

The *vif *in pNL4-3 was replaced with a *vif *from SIVmac, and a recombinant HIV proviral construct pNL-macVif as well as a control pNL-hVif expressing *vif *from HIV-1 was created. We first tested their sensitivity to different human APOBEC3 proteins by a single round HIV replication assay. Although A3B, A3G, and A3H all restricted HIV-1 replication, only A3G was effectively neutralized by *vif *from HIV-1 (Fig. 6A, compare pNL-ΔVif with pNL-hVif). In contrast, *vif *from SIVmac not only more effectively neutralized A3G than *vif *from HIV-1, but also effectively neutralized both A3B and A3H (Fig. 6A). These results are consistent with previous observations.

Next, we used viruses produced from pNL-macVif to infect several different human T cell lines. In a 16-day period, these viruses replicated robustly in both CEM-SS cells and CEM-SS cells expressing an exogenous A3G gene, indicating that they successfully overcame A3G restriction; they replicated well in H9 cells, indicating that they could also overcome A3F and A3DE restriction (Fig. 6B). Notably, their replications in CEM.NKR cell line as well as its clones #5 and #2 were quite similar to the wild-type HIV-1 as seen in Fig. 2B. The relatively permissive clone #5 also supported pNL-macVif viruses replication whereas the non-permissive clone #2 did not. The parental CEM.NKR cell line showed a phenotype between these two clones. These results demonstrated that HIV-1 replication was still restricted in CEM.NKR cells even in the presence of *vif *from SIVmac, which disproved any possible role of A3B and A3H in this HIV-1 restriction.

## Conclusion

In this report, we have not only confirmed the HIV-1 inhibitor in CEM.NKR cells, but also found that it was quite different from any known APOBEC3 proteins. Although CEM.NKR cells expressed A3G and A3F, their activities were effectively neutralized by HIV-1 Vif as evidenced by the decreased G-to-A hypermutations in the wild-type viral genome (Fig. 1D). They, therefore, should not be able to restrict the wild-type HIV-1 replication in CEM.NKR cells. In addition, we found that both A3B and A3H were poorly expressed in CEM.NKR cells (Fig. 3C and data not shown), and importantly, HIV-1 expressing *vif *from SIVmac that effectively inactivated theses two human genes still failed to productively replicate in these cells (Fig. 6B). These results excluded any possible contribution of these APOBEC3 cytidine deaminases to this restriction. Thus, CEM.NKR cells express a novel host factor that potently inhibit HIV-1 replication.

So far, all biologically functional retrovirus restriction factors identified in humans, including A3G/A3F [8,15,16], TRIM5α [28], and CD317 [29], have been counteracted by HIV-1. The A3G/A3F proteins are targeted for proteasomal degradation by Vif [15,16,24,37-39]; the CD317 proteins that can tether viral particles on the cell surface are inactivated by Vpu [29]; unlike old world monkey TRIM5α proteins, human TRIM5α proteins do not have anti-HIV-1 activity. These facts suggest that these host factors have imposed very strong evolutionary pressures during the cross-species transmission of HIV-1 to humans, and the virus needs to develop counteractive mechanisms in order to spread into humans. Why HIV-1 failed to develop a strategy to counteract this novel factor is an intriguing and critical question. One possibility is that it may not be broadly expressed in human tissues, particularly in the primary sites of HIV-1 replication. Among different CEM-derived cell lines, only CEM.NKR cells express this factor. In addition, previous proteomics investigation has found that CEM.NKR cells lose one cellular gene calnexin [31], indicating a difference in gene expression profile between CEM.NKR and its parental cell line. Consistently, our biological analyses demonstrate that CEM.NKR cells express an extra host factor. It is very fortunate that this factor exhibits very strong anti-HIV-1 activity. It will be very interesting to know whether the expression of this factor is broadly inducible in various tissues, particularly in CD4^+ ^T cells and monocytes/macrophages. Such induction could be directly used for HIV-1 suppression. Thus, further characterization of this novel factor and study of its anti-HIV mechanism would lead to the discovery of a powerful antiretroviral treatment.

## Methods

### Plasmids, cell lines, and viruses

Constructions of HIV-1 proviral vectors pNL-Neo, pNL-NeoΔVif, pNL Gag, and pNLΔEnv were described before [30]. Human APOBEC3 expression vectors pcDNA3.1-A3B and A3G and VR-A3H were described before [12,17]. The calnexin gene with a 3' HA tag was inserted into the MuLV expression vector pMSCVneo by EcoRI/XhoI digestion to create pMSCVneo-CANX. pMSCVneo-GFP was described before [7]. To create pNL-hVif and pNL-macVif, pNL4-3 NotI/XbaI was first generated. This plasmid contains a NotI site at the end of *pol *and XbaI site in front of *vpr *and the three ATG codons in *vif *overlapping with *pol *were silenced. After that, the *vif *from SIVmac and HIV-1 were then inserted by NotI/XbaI digestion to create pNL-macVif and pNL-hVif.

The HIV indicator cell line TZM-BI and human T cell lines H9, CEM-SS, CEM-T4, CEM.NKR, and A2.01 were from NIH AIDS Research and Reference Reagent Program. The CEM-SS (A3G) cell line was described before [7]. CEM.NKR clones #1, #2, #3, #4, #5, #6, #7, and #8 were isolated by limiting dilution of CEM.NKR cells in a 96-well culture plate. The HIV-infected H9 and CEM.NKR cell lines were generated by infecting these cells with viruses from pNL-Neo or pNL-Neo Vif followed by G418 selection. CEM.NKR (GFP), CEM.NKR (CANX), CEM-T4 (CANX), and CEM-SS (CANX) cell lines were generated by infecting these cells with viruses from pMSCVneo-CANX or pMSCVneo-GFP followed by G418 selection. T cells were cultured in RPMI 1640 with 10% fetal bovine serum (HyClone). 293T and TZM-BI were cultured in DMEM with 10% bovine calf serum (HyClone).

HIV-1 or MuLV viruses were produced from 293T or Phoenix-AMPHO cells by the standard calcium phosphate transfection.

### Antibodies

The polyclonal anti-human A3G antibody was from W. Greene through the AIDS Research and Reference Reagent Program. The mouse anti-human A3F polyclonal antibody was from Abnova, Taiwan. Other antibodies used included a polyclonal rabbit anti-actin antibody (C-11) (Santa Cruz Biotechnology), PE-conjugated mouse anti-human CXCR4 and FITC-conjugated mouse anti-human CD4 (BD Biosciences), HRP-conjugated anti-HA (Roche Applied Science), and HRP-conjugated anti-rabbit, human, or mouse IgG secondary antibodies (Pierce). Detection of the HRP-conjugated antibody was performed using Supersignal Wetpico Chemiluminescence Substrate kit (PIERCE).

### HIV-1 infection of human T cell lines

A total of 2 × 10^5 ^cells were inoculated with 100 ng p24^Gag ^of HIV-1 viruses at 37°C for three hours. After removal of the inocula followed by three times of extensive washing, cells were cultured in 24-well plates. Culture supernatants were then collected daily for measurement of p24^Gag ^by ELISA.

### Sequencing of viral genes

Total cellular DNAs were extracted from HIV-infected H9 and CEM.NKR cells by the DNeasy tissue kit (Qiagen). A 420-bp fragment was PCR-amplified by a previously described primer pair [16] and cloned into the pCR4-TOPO vector (Invitrogen). Multiple clones were selected and sequenced by the flanking T3 and T7 primers.

### Real-time PCR measurement of viral reverse transcripts

A3DE, A3H, or GAPDH (Glyceraldehyde 3-phosphate dehydrogenase)-specific PCR primer pairs as well as an A3H-specific fluorescence-labeled probe were described previously [12,17]. The sequence of A3DE-specific fluorescence-labeled probe is 5'-/56-FAM/CGCTCAAATCTCCTTTGGGACACAGG/36-TAMSp/-3'. Total cellular RNAs were extracted by TRIzol (Invitrogen) from different cell lines. 1 μg of total RNA was subjected to reverse transcription using Superscriptase II reverse transcriptase and oligo(dT)12–18 as a primer (Invitrogen). A3DE and A3H mRNAs were first determined by TaqMan^® ^Master Mix gene expression kit (Applied Biosystems) and then normalized to the levels of GAPDH mRNA, which were determined by the SYBR Green^® ^PCR Master Mix kit (Applied Biosystems).

### Heterokaryon formation

A previously described protocol was used [30]. Briefly, 293T cells were seeded in six-well plates at 8 × 10^5^/well in 2 ml medium. Twelve hours later, cells were transfected with 6 μg of HIV Env expression vector pNLΔGag and washed with PBS four hours' later. Simultaneously, 8 × 10^5 ^T cells were infected with 500 ng of VSV-pseudotyped Env-defective HIV-1 from pNL Env-transfected 293T cells at 37°C for three hours. After removal of the inocula and extensive washing, infected T cells were added to the Env-expressing 293T cell culture. After 48 hours, supernatants from these co-cultures were collected to infect TZM-BI cells. Viral infectivity was finally determined by measuring cellular luciferase activities after another 48 hours.

## Competing interests

The authors declare that they have no competing interests.

## Authors' contributions

YH, TZ, and YHZ designed the study. TZ, YH, YD, and XW performed experiments. YH, TZ, and YHZ analyzed the data, and YHZ wrote the paper.
